# Effect of the rest interval duration between contractions on muscle fatigue

**DOI:** 10.1186/1475-925X-11-89

**Published:** 2012-11-26

**Authors:** Daniel V Nogueira, Sidney B Silva, Luiz Carlos de Abreu, Vitor E Valenti, Mahmi Fujimori, Carlos Bandeira de Mello Monteiro, Charli Tortoza, Wellington Ribeiro, Rodrigo A Lazo-Osório, Carlos J Tierra-Criollo

**Affiliations:** 1 Universidade do Vale do Paraíba (UNIVAP)/Grupo de Instrumentação e Processamento de Sinais (GIPSI), IP&D, Rua Tertuliano Delphim Jr, 181, São José dos Campos, São Paulo, 12246-080, Brazil; 2 Laboratório de Delineamento de Estudos e Escrita Científica, Departamento de Morfologia e Fisiologia, Faculdade de Medicina do ABC, Av. Príncipe de Gales, 821, Santo André, São Paulo, 09060-650, Brazil; 3 Escola de Artes, Ciências e Humanidades da Universidade de São Paulo, Av. Arlindo Béttio, 1000 Ermelino Matarazzo, São Paulo, SP, CEP: 03828-000, Brazil; 4 Programa de Pós-Graduação em Fisioterapia, Faculdade de Ciências e Tecnologia, Universidade Estadual Paulista, Rua Roberto Simonsen, 305, Presidente Prudente, São Paulo, 19060-900, Brazil; 5 Programa de Engenharia Biomédica - COPPE, Universidade Federal do Rio de Janeiro (UFRJ), Av. Pedro Calmon, 550 - Prédio da Reitoria, 2° andar, Rio de Janeiro, RJ, 21941-901, Brazil

**Keywords:** Fatigue, Elbow, Muscles, Torque, Rest interval

## Abstract

**Background:**

We aimed to investigate the effect of rest interval, between successive contractions, on muscular fatigue.

**Methods:**

Eighteen subjects performed elbow flexion and extension (30 repetitions) on an isokinetic dynamometer with 80º of range of motion. The flexion velocity was 120º/s, while for elbow extension we used 5 different velocities (30, 75, 120, 240, 360º/s), producing 5 different rest intervals (2.89, 1.28, 0.85, 0.57 and 0.54 s).

**Results:**

We observed that when the rest interval was 2.89 s there was a reduction in fatigue. On the other hand, when the rest interval was 0.54 s the fatigue was increased.

**Conclusions:**

When the resting time was lower (0.54 s) the decline of work in the flexor muscle group was higher compared with different rest interval duration.

## Background

Muscle fatigue has been received attention in the literature. There is a consensus that defines it as the inability of muscles to produce force (torque) with the same efficiency over a period of time or after repeated contractions [1]. Although this matter has been investigated several times in the scientific community, there is the need to understand some processes that are relevant to this mechanism.

Recent studies [1-3] measured muscle fatigue based on new technologies and also based on the development of the isokinetic dynamometer. The dynamometer is able to measure the muscle torque in particular joints and it also limits the speed and range of motion [4]. It was showed that the torque produced in the joint decreases after multiple repetitions of the same movement. This fact characterizes the muscle fatigue [4].

The protocols performed to measure muscle fatigue through the isokinetic dynamometry consider the speed, amplitude, time of execution of the movement and the rest interval between sets [1,2,5,6]. These protocols have used a single rest interval duration for the movements of flexion and extension. However, it is not clear in the literature the involvement of this equipment in the elbow flexor muscle group. Therefore, we aimed to investigate the effect of different rest intervals duration on fatigue in the elbow flexor muscle group.

## Methods

### Subjects

We analyzed 18 male non-athlete subjects, between 18 and 35 years old (160 cm-180 cm). The subjects presented no musculoskeletal dysfunction and were able to perform all procedures. Each subject signed a consent letter and the study was approved by the Ethics Committee in Research of Universidade do Vale do Paraiba (protocols number: 003/2008).

### Instruments

To collect data related to the work produced by the elbow flexor and extensor muscles, we used a Computed Isokinetic, Biodex Multi-Joint System 3 model (Biodex Medical System Inc) and its accessories for the elbow joint evaluation. This equipment is connected to a computer with specific software, which allows view, acquisition and registration of several variables.

### Procedures

Each volunteer performed six tests in the isokinetic dynamometer in different days. The rate of extension ranged randomly at 30º/s, 75º/s, 120º/s, 240º/s and 360º/s, totaling five tests (only in the dominant limb). The first test performed on each subject was used as an adaptation apparatus and was discarded. The range of motion in all protocols used was 80° for elbow flexion and extension. The rest interval between the tests was between 72 hours and 96 hours. All attempts were preceded by warming up the elbow flexor and extensor muscle groups with 2.0 kg dumbbell of 20 repetitions for each movement. After warm up, all subjects performed stretching exercises for 30 seconds of specific muscle groups assessed. They were seated, fixed to the dynamometer through tracks in the region of the trunk and had the dominant upper limb coupled to an accessory that allowed the evaluation of elbow flexion and extension.

Before sampling, the volunteers performed three to five submaximal repetitions to recognize the type of exercise. During the tests the subjects received verbal encouragement to perform maximum effort with visual feedback. The verbal encouragement was based on the following sentences for all subjects: “Go ahead!” and “Stronger!”

The following variables exported for analysis using the Matlab software from MathWorks: peak torque, time and range of motion produced by the flexor and extensor muscles of the elbow.

#### Data analysis

Muscle fatigue was estimated by the value of the slope, obtained by linear regression applied to the values of work over the 30 contractions. The linear regression was considered as an approach to modeling the relationship between a scalar dependent variable y and another explanatory variable denoted X.

The work performance was considered as an action on a body so that there is a displacement of the point of application, however small, in the direction of the force. Thus a force does work when there is movement under the action of the force.

Torque was considered as the tendency of a force to rotate an object about an axis, fulcrum, or pivot.

The rest interval duration between contractions were obtained by marking the beginning and end of each movement, and the number of samples in this range was multiplied by 0.010 s considering the frequency of data acquisition (100 Hz). For comparison, it was calculated theoretical rest interval duration given by:

(1) Δt=Δθϖ,

Δ*θ=80*° and ϖ is the selected angular speed (°/s). The time difference between the theoretical and obtained time series was calculated by means of the relative error.

### Statistical analysis

We considered the angular coefficient as the value of the tangent of the angle alpha that the line makes with the x-axis. Standard statistical methods were used for the calculation of means and standard deviations. Normal Gaussian distribution of the data was verified by the Shapiro-Wilk goodness-of-fit test (z value >1.0). Considering that all distributions were non-parametric, we applied the nonparametric Wilcoxon test for comparison of the angular coefficients obtained in each protocol. A significance level of p<0.05 was adopted.

## Results

Table 1 shows the different speeds for the rest interval duration between contractions of the flexor muscle group-time series obtained from the torque. As expected, the standing time between consecutive elbow flexion decreased while the speed increased. We noted that the relative error between the theoretical value of the interval and the time series obtained in the torque increased with speed. It is further noted in Table 1 that the relative error between the theoretical value of the interval and the time series obtained for the torque is increased with the extension speed. The rest interval duration between successive extensions averaged 0.85 ± 0.04 s.

**Table 1:** **Time interval** (**rest**) **between each contraction of the flexor muscle group**

***ϖ *(**^**o**^**/s)**	**Temporal series of the torque (s)**	**Standard deviation**	**Theoretical (s)**	**Relative error (%)**
30/120	2.89	0.0549	2.67	6.7
75/120	1.28	0.0467	1.07	15.7
120/120	0.85	0.0426	0.67	21.2
240/120	0.57	0.0488	0.33	42.1
360/120	0.54	0.0759	0.22	59.2

**Figure 1:**
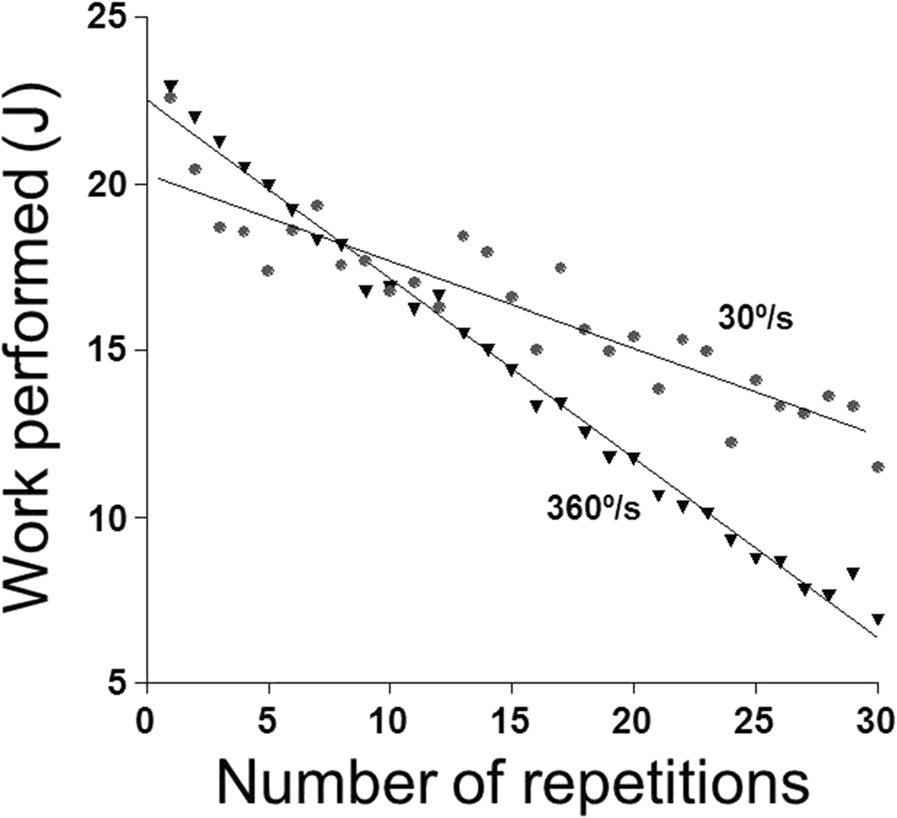
Values of work performed in one flexor muscular group, with several extension speeds in one subject.

Figure 1 illustrates the relationship between the work performed in each contraction in one volunteer. We noted that for both variables the slow speed of extension (30°/s) produced an average of 2.86 s rest between each flexion. Regarding the faster one (360°/s) at time average of 0.54 s we observed a significant decrease of the work in the flexor muscle group. These findings were observed in all subjects, indicating that all protocols with varying speeds of elbow extension were enough to induce fatigue.

**Figure 2:**
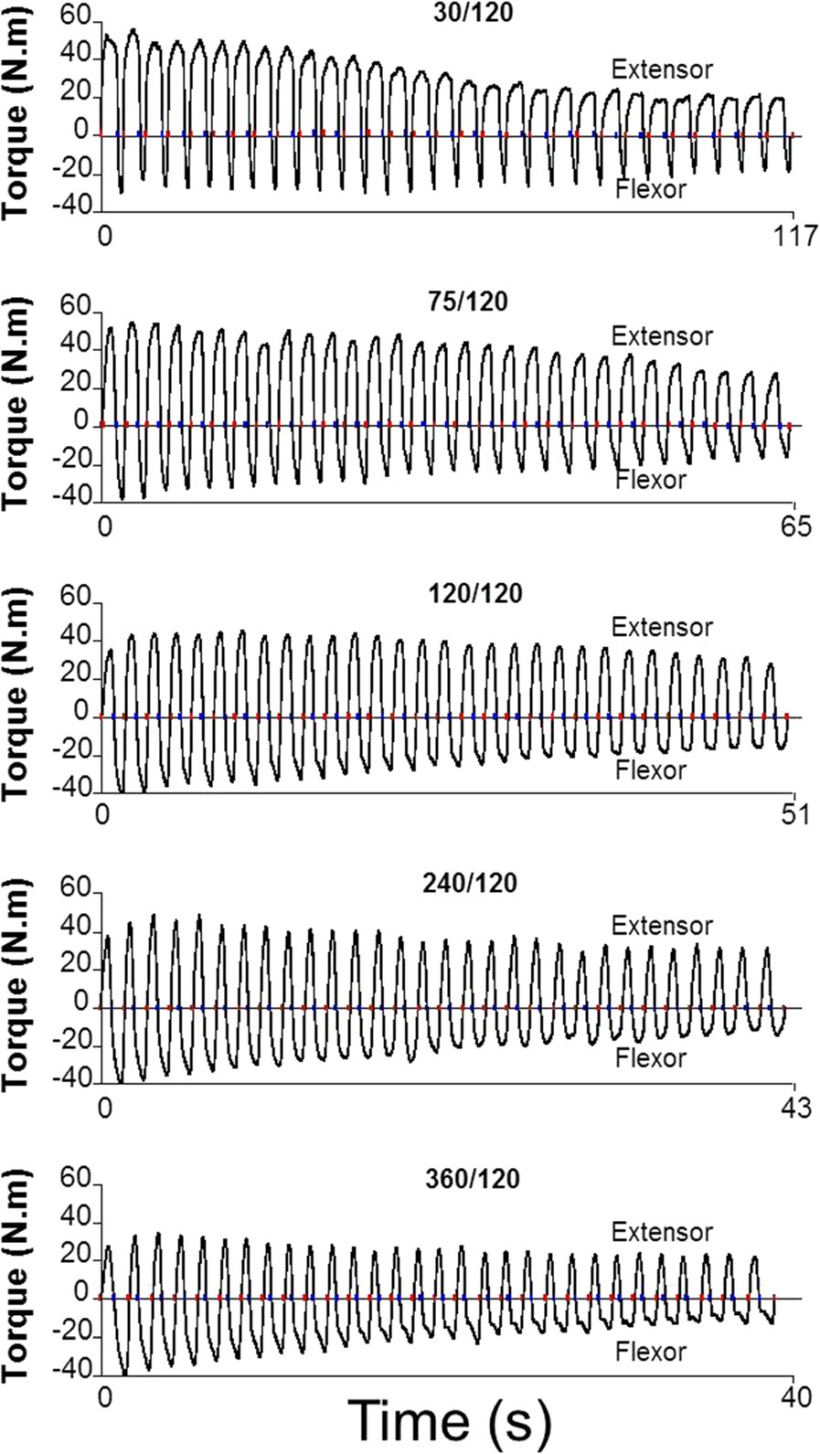
**Aligned time series values of flexor and extensor elbow muscle torques in 5 different attempts (30, 75, 120, 240 and 360º/s) of one volunteer.** The colored dots indicate the beginning and end of each movement.

Figure 2 displays the time series obtained from different attempts, by varying the extension speed. We observed that the lower extension speed (between flexion) was smaller than the reduction observed during flexion.

**Figure 3:**
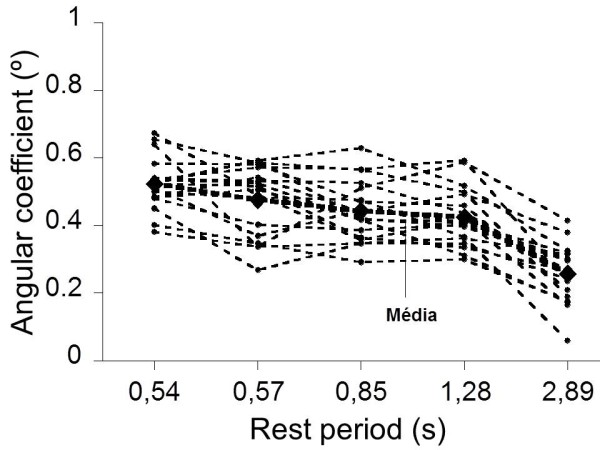
**Variation of the angular coefficients (º) obtained from the momentum generated by the flexor muscles in different rest intervals.** The darker line represents the average of the slopes of all subjects.

The coefficients obtained for the slower rate of extension (longer rest intervals) were significantly lower than other speeds (p < 0.016) (Table 2). Moreover, when the rate of extension was 360°/s (lower resting time), the angular coefficients presented significant difference (p < 0.05) compared to other speeds. Among the intermediate speed (75°/s, 120°/s and 240°/s), we did not observe statistically significant differences (p> 0.05) (Figure 3).

**Table 2:** **P values regarding the comparison of the angular coefficient** (**º**)

***ϖ *(**^**o**^**/s)**	**30/120**	**75/120**	**120/120**	**240/120**
**75/120**	p=0.008	–	–	–
**120/120**	p=0.008	p=0.742	–	–
**240/120**	p=0.016	p=0.195	p=0.383	–
**360/120**	p=0.008	p=0.016	p=0.039	p=0.05

**Figure 4:**
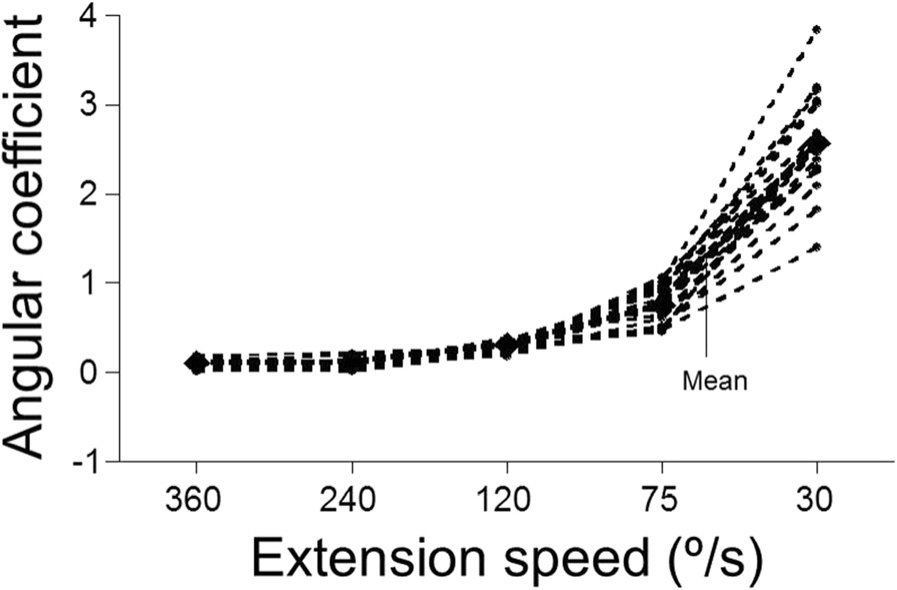
**Variation of the angular coefficients (º) obtained from the momentum generated by the extensor muscles at different speeds.** The darker line represents the average of the slopes of all subjects.

According to Figure 4, regarding the extensor muscle group, the behavior of the angular coefficients in different extension speeds followed a different pattern. When extension speed was 30°/s the angular coefficients averaged 2.57º, indicating large inclination and intense fatigue. With respect to the 360º/s, the mean coefficient was 0.07º, indicating small fatigue.

## Discussion

Our study was undertaken to evaluate the effects of different rest intervals duration on fatigue of the elbow flexor muscle group. Based on our results, when the rest interval between each contraction was higher (mean 2.89 s), the performance was lower compared with other intervals time (1.28 s, 0.85 s, 0.57 s to 0.54 s). According to Fitts (1996) [7], during the standing time after muscle fatigue, there is a fast recovery of the fibers which becomes slower and may take an hour or longer to return to the pre-fatigue state. It was showed that muscle fatigue presents an important factor in sports performance. In addition, it may be responsible for various musculoskeletal disorders [3], since it influences proprioception and neuromuscular control [8]. Therefore, understanding the process of muscle fatigue is very important for professionals in the rehabilitation and physical training area.

Basically, the mechanism involved in muscle fatigue begins with a precipitous drop in the capacity to develop muscle tension, generating the drop development [7]. The entire mechanism of muscle fatigue is reversible with time. For this reason many authors use protocols with rest intervals between sets of exercises. Parcell et al. (2002) [5] found that many authors fail to mention the rest interval duration used between sets proposed mentioning only that "adequate rest was kept" and others simply use a “relatively high” to ensure recovery. Parcell et al. (2002) [5] reported that the minimum time required for recovery of muscle contractions after four concentric isokinetic at speeds of 60, 120, 180, 240 and 360º/s is 60 s. However, all of these protocols did not take into account the existing recovery time when the antagonist muscles contract, i.e. the rest interval duration between contractions. The results presented in our study showed that it is possible to control the rest interval duration between successive movements through the isokinetic dynamometer. When using this equipment, it is possible to limit the amplitude and speed of movement making it relatively constant [4]. Thus, it is also possible to standardize the time of rest between each contraction.

Regarding the results obtained for the rest interval duration between successive movements of elbow flexion, it was noted that it approached the theoretical values at speeds of 30, 75 and 120°/s, a tendency, as expected, to be slightly higher, while in the speeds of 240 and 360º/s the rest interval were more distant than the theoretical values. It was previously showed that the set speed in isokinetic dynamometers does not remain constant throughout the range of motion, tending to present a larger variation at higher speeds [9]. Regardless of the rest time range in each experimental condition it may be noticed that all protocols were sufficient to generate fatigue of elbow flexor muscle group. These results agree with the findings of other authors [2,3], which demonstrated that even using a protocol with fewer repetitions there is also a decrease in the muscle work.

It is interestingly to note that when the rest time between successive flexion changed we also observed changes in the total effort time, since the number of repetitions was the same for all protocols. Thus, due to the reduction in extension speed the rest interval between the flexion movements is higher and, as a consequence, the total duration of the task also increases. Similarly, when the rest time is lower the entire task becomes reduced.

As noted, the flexor and extensor torques followed a different pattern during the 30 repetitions and this result may be due to some variables. For example, when the extension speed was slower (30°/s) the torque extensor declined considerably whereas the flexor muscles in the same trial showed slight decrease in torque. As soon as the extension speed increased the reduction of the extensor muscle torque was attenuated while the flexor muscle group was increased. We may suggest that the behavior of the extensor muscle torque was slightly influenced by the rest interval between contractions because this was almost constant as a function of the flexion speed, which was 120°/s. Nonetheless, it was influenced with higher intensity by the change of speed in some protocols. The behavior of flexor muscle torque was slightly influenced by the flexor speed and was influenced with higher intensity by the rest time between movements.

The dynamometers provide fatigue indices by means of calculations based on of the work generated at different stages of the task [10]. However, there are some limitations in using this type of analysis, for example, it does not consider the effort across the task at hand, or even the lack of view of how the software is processing the signal. Furthermore, it was noted that this type of analysis presents a low correlation coefficient interclass [6]. Some authors have previously estimated the muscle fatigue by varying the speed of movement in relation to time during the activity carried out in isotonic contractions [11]. Others found it necessary to calculate it by the fall of the torque generated or even the total work done (full torque in relation to the displacement) [1,12].

It has been shown that muscle fatigue (decline in work) presents an important relationship with the rest time between exercises performance [2]. As noted by some authors, the rest interval duration between the series in a protocol, when reduced, may increase fatigue [2], [5], [10]. Nevertheless, these protocols have not taken into account the resting period of the agonist muscles when the antagonist muscles were also involved during the motion. In this study, we observed that when the rest interval between each contraction of the flexor muscle group (time of antagonist muscles contraction) was higher (mean 2.89 s), the reduction of work was lower when compared with other rest intervals (1.28 s, 0.85 s, 0.57 s to 0.54). According to Fitts [7], during the time after muscle fatigue, there is a fast recovery of the fibers, which becomes slower and may take an hour or longer to return to the state of pre-fatigue. It is suggested that the time of 2.89 s in our experimental condition was sufficient for rapid muscle recovery, however, it was interrupted by reapplication of the muscles.

## Conclusions

When the rest time was lower (mean 0.54 s) the decline of work in the elbow flexor muscle group was higher compared with different rest times. It is suggested that the fast recovery of the fibers was prematurely interrupted. Clarifying the relationship of the rest interval duration after strenuous exercise on muscle performance may certainly improve methods for training and physical rehabilitation.

## Competing interests

The authors declare that they have no competing interests.

## Authors’ contributions

DVN, SBS, LCA, MF, CBMM, VEV, CT, WR, RALO and CJTC participated in the acquisition of data and revision of the manuscript. VEV, CBMM, MF and LCA determined the design and performed the statistical analysis. All authors conceived the study, interpreted the data and drafted the manuscript. All authors read and gave final approval for the version submitted for publication.
